# Clinical Phenotype and Disease Course of Inflammatory Bowel Disease in Iran: Results of the Iranian Registry of Crohn’s and Colitis (IRCC)

**DOI:** 10.34172/aim.2024.27

**Published:** 2024-04-01

**Authors:** Bahar Saberzadeh-Ardestani, Amir Ali Khosravi, Fariborz Mansour-Ghanaei, Homayoon Vahedi, Nadieh Baniasadi, Mohammadreza Seyyedmajidi, Baran Parhizkar, Ahmad Hormati, Sayed Jalalledin Naghshbandi, Somaieh Matin, Amir Abbas Hassan Zadeh, Tarang Taghvaei, Mohsen Bahrami, Mandana Rafeey, Mitra Ahadi, Hassan Vossoughinia, Hashem Muosavi, Shahsanam Gheibi, Roya-Sadat Hosseini-Hemmatabadi, Abbas Yazdanbod, Saied Matinkhah, Farshad Sheikh Esmaeili, Hafez Fakheri, Seyed Hamid Moosavy, Iradj Maleki, Siavosh Nasseri-Moghaddam, Bardia Khosravi, Fatemeh Farahmand, Mehri Najafi, Hosein Alimadadi, Masoud Malekzadeh, Amir Anushiravani, Amir Kasaeian, Sudabeh Alatab, Anahita Sadeghi, Amir Reza Radmard, Shadi Kolahdoozan, Zeynab Rajabi, Ali Reza Sima

**Affiliations:** ^1^Digestive Disease Research Center, Digestive Disease Research Institute, Tehran University of Medical Sciences, Tehran, Iran; ^2^Gastrointestinal and Liver Diseases Research Center, Guilan University of Medical Sciences, Guilan, Iran; ^3^Noncommunicable Diseases Research Center, Bam University of Medical Sciences, Bam, Iran; ^4^Golestan Research Center of Gastroenterology, Golestan University of Medical Science, Gorgan, Iran; ^5^Liver and Digestive Research Center, Research Institute for Health Development, Kurdistan University of Medical Sciences, Sanandaj, Iran; ^6^Department of Internal Medicine, School of Medicine, Lung Diseases Research Center, Ardabil, Iran; ^7^Digestive Diseases Research Center, Imam Khomeini Hospital, Ardabil University of Medical Sciences, Ardabil, Iran; ^8^Nazeran Hospital, Mashhad, Iran; ^9^Gut and Liver Research Center, Non-communicable Diseases Institute, Mazandaran University of Medical Sciences, Sari, Iran; ^10^Sasan Alborz Biomedical Research Center, Masoud Gastroenterology and Hepatology Center, Tehran, Iran; ^11^Liver and Gastrointestinal Diseases Research Center, Tabriz University of Medical Sciences, Tabriz, Iran; ^12^Department of Gastroenterology and Hepatology, Mashhad University of Medical Sciences, Mashhad, Iran; ^13^Maternal and Childhood Obesity Research center, Urmia University of Medical Sciences, Urmia, Iran; ^14^Shahid Sadoughi University, Yazd, Iran; ^15^Digestive Disease Research Center, Ardabil University of Medical Science, Ardabil, Iran; ^16^Isfahan University of Medical Sciences, Isfahan, Iran; ^17^Shahid Mohammadi Hospital, Hormozgan University of Medical Sciences, Bandar Abbas, Iran; ^18^Pediatric Gastroenterology and Hepatology in Children Research Center, Tehran University of Medical Sciences, Tehran, Iran; ^19^Tehran University of Medical Sciences, Tehran, Iran; ^20^Department of Pediatrics, Tehran University of Medical Sciences, Tehran, Iran; ^21^Digestive Oncology Research Center, Digestive Diseases Research Institute, Shariati Hospital, Tehran University of Medical Sciences, Tehran, Iran; ^22^Research Center for Chronic Inflammatory Diseases, Shariati Hospital, Tehran University of Medical Sciences, Tehran, Iran; ^23^Clinical Research Development Unit, Shariati Hospital, Tehran University of Medical Sciences, Tehran, Iran; ^24^Department of Radiology, Shariati Hospital, Tehran University of Medical Sciences, Tehran, Iran; ^25^Department of Internal Medicine, Shariati Hospital, Tehran University of Medical Sciences, Tehran, Iran

**Keywords:** Clinical phenotype, Disease course, Inflammatory bowel disease

## Abstract

**Background:**

Data on the epidemiology of inflammatory bowel disease (IBD) in the Middle East are scarce. We aimed to describe the clinical phenotype, disease course, and medication usage of IBD cases from Iran in the Middle East.

**Methods:**

We conducted a cross-sectional study of registered IBD patients in the Iranian Registry of Crohn’s and Colitis (IRCC) from 2017 until 2022. We collected information on demographic characteristics, past medical history, family history, disease extent and location, extra-intestinal manifestations, IBD medications, and activity using the IBD-control-8 questionnaire and the Manitoba IBD index, admissions history, history of colon cancer, and IBD-related surgeries.

**Results:**

In total, 9746 patients with ulcerative colitis (UC) (n=7793), and Crohn’s disease (CD) (n=1953) were reported. The UC to CD ratio was 3.99. The median age at diagnosis was 29.2 (IQR: 22.6,37.6) and 27.6 (IQR: 20.6,37.6) for patients with UC and CD, respectively. The male-to-female ratio was 1.28 in CD patients. A positive family history was observed in 17.9% of UC patients. The majority of UC patients had pancolitis (47%). Ileocolonic involvement was the most common type of involvement in CD patients (43.7%), and the prevalence of stricturing behavior was 4.6%. A prevalence of 0.3% was observed for colorectal cancer among patients with UC. Moreover,15.2% of UC patients and 38.4% of CD patients had been treated with anti-tumor necrosis factor (anti-TNF).

**Conclusion:**

In this national registry-based study, there are significant differences in some clinical phenotypes such as the prevalence of extra-intestinal manifestations and treatment strategies such as biological use in different geographical locations.

## Introduction

 Inflammatory bowel disease (IBD) is a chronic, relapsing inflammation of the gastrointestinal tract and comprises ulcerative colitis (UC) and Crohn’s disease (CD). The etiology of the disease is not fully known, but multiple genetic and environmental factors influence the pathogenesis and course of the disease.

 The prevalence of IBD has been rising during the last decades,^1,2^ with five million patients affected worldwide.^3^ The highest incidence of IBD is reported in Northern Europe and Northern America.^4^ It is estimated that 0.1% - 0.2% of the population in Japan and South Korea may have IBD compared to 0.5% of the population in the United States.^5^ Although IBD was considered a disease in Westernized countries, the incidence of the disease is increasing in Asia.^6-9^ Among the reported incidence of IBD, Japan and South Korea had the highest rate of 14.2 and 9 per 100 000 individuals, respectively.^10,11^ Other Asian countries have lower incidence rates with various stages of industrialization.^12,13^

 There are several notable differences among the demographics of IBD patients in Asia and the West.^14^ For instance, data from Asia supports male dominancy for CD compared to female dominancy in the West. The age distribution of patients in Western countries consists of two peaks: one at 20 to 30 for CD and 30 to 40 years for UC, and the other at 60 to 70 for both diseases.^15^ Reports from Asia show the first peak with a much less prominent second peak.^16^

 Clinical presentations of UC patients are comparable between Asian countries and the West, but more differences have been reported for CD patients. Regarding disease location, the Western literature demonstrates relatively equal proportions of CD patients with ileal, colonic, or ileocolonic involvement. However, ileocolonic involvement is the most common phenotype observed in 50-70% of CD patients in East Asia.^17-20^ Another disparity between the East and the West is the higher prevalence of perianal disease and lower colectomy rate in East Asia.^14^

 The prevalence of IBD is rising in Asia concurrently with industrialization. Heterogeneity in the genetic compound and environmental risk factors has led to discrepancies in age at diagnosis, clinical manifestations, and complications when comparing Eastern and Western countries. Most of the data concerning the epidemiology of IBD in Asia originated from the Eastern population, and there are limited studies conducted in the Middle East. In this study, we reported the natural history of IBD in Iran and showed IBD behavior, clinical outcome, and medication usage as a representative population of the Middle East. Exploring the natural history of IBD may help address social burdens and pathogenesis and inform policy decision-making to improve disease management.

## Materials and Methods

###  Study Design and Participants

 This is a cross-sectional study of the natural history of IBD in Iran. We used data from the Iranian Registry of Crohn’s and Colitis (IRCC), a national registry with at least one center in each province of Iran.^21^ We included all patients with confirmed IBD diagnoses from December 2017 to July 2022. We followed the international guidelines for IBD diagnosis based on clinical, radiological, colonoscopic, and pathologic findings.^22^ The methodology for data collection in IRCC is described in detail in previous studies.^21^

 After obtaining informed consent from the patients, data regarding the specifications of the disease was recorded by a gastroenterologist (IRCC collaborators), including IBD subtype (CD or UC), age at diagnosis, duration of the disease (interval between age at diagnosis and current age), UC extent based on Montreal classification (categorized to proctitis, left-sided colitis, and pancolitis),^23^ CD location (classified as ileal, colonic, ileocolic, and upper GI), CD behavior (including inflammatory, fistulizing, stricture forming), history of colon cancer, IBD related surgeries, and extra-intestinal manifestations (including sclerosing cholangitis [PSC], ankylosing spondylitis [AS], autoimmune hepatitis [AIH], erythema nodosum, uveitis, pyoderma gangrenosum [PG], and peripheral arthritis).^21^

 Other general information was obtained through a telephone interview with a research assistant (registrar). The gathered information comprised demographic data, comorbidities, educational background, family history of IBD (including the degree of the affected relative and number of family members with IBD), the disease activity during the past two weeks using the IBD-control-8 questionnaire (with a score above 13 defined as inactive disease),^24^ disease activity during the past six months using the Manitoba IBD Index (with a score above four defined as inactive disease),^25^ IBD medications (consisting of prednisolone, 5-aminosalicylic acid [5-ASA], immunomodulators, antitumor necrosis factors [anti-TNF]), emergency room visits in the past 12 months, and admissions in the past three months.^21,26^

 Case enrolment was based on the diagnoses of gastroenterologists who worked with the IRCC, and were committed to using standard illness definitions and protocols. The quality of data collection was checked by registrars randomly recording and reviewing interviews. Additionally, our software’s architecture includes validation rules that prevent incorrect data from being registered, as well as a monitoring dashboard that allows the executive management to track response times and missing data. Every registrar received training at the IRCC office. To assess the construct validity of clinician-reported questions, there was also a process for randomly testing physician-answered questions.

 Statistical analysis was performed using Stata 11.2 (Stata Corp. 2011, Stata Statistical Software, Release 12, College Station, TX, Stata Corp LP) for Windows. Categorical data are depicted as percentage. Continuous variables are reported as median and first and third interquartile range (IQR).

## Results

 A total of 9746 patients with confirmed IBD diagnosis were registered in IRCC at the time of writing this paper. Of those, 7793 patients had UC and 1953 patients had CD. The UC to CD ratio in our study cohort was 3.99.

###  UC Clinical Characteristics

Table 1 shows the clinical characteristics and demographic data of UC patients. The male to female ratio was 1.1 in UC patients. The median age at diagnosis was 29.2 (IQR: 22.6,37.6). The mean duration of the disease was 7.4 years. A first- or second-degree family member with IBD was reported by 17.9% of patients, with 10.6% having a first-degree relative with IBD. Persian (59.7%) and Azeri (17%) were the most common ethnicities, followed by Kurd (8.8%), Lur (3%), and others (11.4%), such as Arab and Turkmen.

**Table 1:** Clinical Characteristics and Demographic Features of IBD Patients

**Variables**^a,b^	**IBD**	
	**UC (N=7793)**	**CD (N=1953)**
Age, No. (%)		
0-9	24 (0.3)	2 (0.2)
10-19	156 (2.0)	83 (4.3)
20-29	1090 (14.1)	345 (17.9)
30-39	2573 (33.4)	601 (31.1)
40-49	1928 (25.0)	447 (23.2)
50-59	1170 (15.2)	259 (13.4)
60-69	520 (6.7)	136 (7.0)
70-79	195 (2.5)	47 (2.4)
80-89	53 (0.7)	10 (0.5)
90-99	3 ( < 0.1)	0 (0.0)
Gender, n (%)		
Female	3718 (47.7)	855 (43.8)
Male	4075 (52.3)	1098 (56.2)
Education, n (%)		
Illiterate	246 (3.1)	44 (2.3)
Primary school	693 (8.9)	134 (6.9)
Middle school	830 (10.7)	230 (11.8)
High school	2222 (28.5)	552 (28.3)
Associate degree	534 (6.9)	136 (7.0)
Bachelor	2192 (28.1)	546 (28.0)
Master	845 (10.8)	238 (12.2)
Doctoral	231 (3.0)	73 (3.7)
Ethnicity, n (%)		
Persian	4651 (59.7)	1324 (67.8)
Azeri	1323 (17.0)	254 (13.0)
Lur	236 (3.0)	68 (3.5)
Kurd	687 (8.8)	114 (5.8)
Arab	74 (0.9)	10 (0.5)
Turkmen	56 (0.7)	16 (0.8)
Other	766 (9.8)	167 (8.5)
Years of Follow-up, Mean (SD)	7.4 (6.6)	7.6 (6.7)
Familial cases, n (%)		
1^st^ degree	823 (10.6)	198 (10.1)
2^nd^ degree	514 (6.6)	117 (6.0)
1^st^ and 2^nd^ degree	61 (0.8)	8 (0.4)
Number of patients in family, n (%)		
1	1249 (16.0)	302 (15.46)
2	135 (1.7)	20 (1.0)
≥ 3	14 (0.2)	1 (0.05)
Comorbidities		
Anemia	1846 (23.7)	585 (29.9)
Low back pain	664 (8.5)	192 (9.8)
HTN	587 (7.5)	152 (7.8)
Pulmonary disease	161 (2.1)	59 (3.0)
Diabetes Mellitus	302 (3.9)	80 (4.1)
Renal Disease	310 (4.0)	86 (4.4)
Liver Disease	637 (8.2)	111 (5.7)
Depression	457 (5.9)	138 (7.1)
Cancer	41 (0.5)	25 (1.3)
TB	14 (0.2)	0.7 (0.4)
HBV	21 (0.3)	6 (0.3)

IBD, inflammatory bowel disease; UC, ulcerative colitis; CD, Crohn’s disease. ^a^Percentages do not include missing values and were calculated for each row by dividing on the corresponding N value. ^b^Percentages from each subcategory may not add up to the exact number of total reported cases due to missing values and/or non-mutually exclusive variables.

###  UC Behavior and Clinical Outcome

 The distribution of age at diagnosis had one peak at 30-40 years. Most patients had extensive disease, with pancolitis in 47% (95% CI: 45-49%) of cases. The rate of extra-intestinal manifestations was 5.2% (95% CI: 4.7-5.7%). Colectomy and colorectal cancer history were found in 2.7% (95% CI: 2.3-3.1%) and 0.3% (95% CI: 0.2-0.4%) of patients, respectively. The most common treatment was 5-ASA (93.5%, 95% CI: 93-94%), followed by immunomodulators, prednisolone, and anti-TNF. Among immunomodulator users, 96.2% received azathioprine, 2.8% received 6-mercaptopurine, and 1% received methotrexate. Among anti-TNF users, 68.8% received adalimumab (CinnoRA®), and 31.2% received infliximab (Remicade®). Table 2 shows IBD patients’ phenotype, disease course, and outcome.

**Table 2:** Clinical Phenotype, Disease Course and Outcomes of IBD Patients

**Variables**^a,b^	**IBD**	
	**UC (N=7793)**	**CD (N=1953)**
Age at diagnosis, Median (IQR)	29.2 (22.6,37.6)	27.6 (20.6,37.6)
Extra-intestinal Manifestations, n (%)	408 (5.2)	70 (3.6)
Extent, n (%)		
Proctitis	681 (18.9)	N/A
Left-sided colitis	1224 (34.1)	N/A
Pancolitis	1689 (47.0)	N/A
Location, n (%)		
Ileal	N/A	267 (34.6)
Colonic	N/A	160 (20.8)
Ileocolonic	N/A	337 (43.7)
Upper GI	N/A	7 (0.9)
Disease behavior, n (%)		
Fistulizing or penetrating	N/A	212(10.9)
Stricturing	N/A	90 (4.6)
Non stricturing or fistulizing	N/A	1651 (84.5)
IBD medication, n (%)		
Prednisolone	3288 (42.2)	993 (50.8)
5-ASA	7288 (93.5)	1620 (82.9)
Immunomodulator	3438 (44.1)	1206 (61.8)
Anti-TNF	1188 (15.2)	750 (38.4)
Active disease during the past 2 weeks, n (%)	4966 (63.7)	1088 (55.7)
Active disease during 6 months, n (%)	1359 (17.4)	493 (25.2)
ER visits in the past 12 months, n (%)	986 (12.7)	276 (13.1)
Admissions in the past 3 months, n (%)	778 (10.0)	299 (15.3)
History of colon cancer, n (%)	21 (0.3)	10 (0.5)
IBD-related surgeries, n (%)	209 (2.7)	278 (14.2)

IBD, inflammatory bowel disease; UC, ulcerative colitis; CD, Crohn’s disease; ER, emergency room; 5-ASA, 5-aminosalicylic acid; Anti-TNF, anti-tumor necrosis factor. ^a^Percentages do not include missing values and were calculated for each row by dividing on the corresponding N value. ^b^Percentages from each subcategory may not add up to the exact number of total reported cases due to missing values and/or non-mutually exclusive variables.

###  CD Clinical Characteristics

Table 1 shows the clinical characteristics and demographic data of CD patients. The male-to-female ratio was 1.28. The mean disease duration was 7.6 years. A first- or second-degree family member with IBD was reported by 16.5% of patients, with 10.1% having a first-degree relative with IBD. CD patients comprised Persian (67.8%), Azeri (13%), Kurd (5.8%), Lur (3.5%), and other ethnicities (9.8%).

###  CD Behavior and Clinical Outcome

 The median age at diagnosis was 27.6 (IQR: 20.6,37.6) in CD patients. The distribution of age at diagnosis had one peak at 30-40 years. Ileocolonic involvement was the most common location (43.7%, 95% CI: 40.6-47.6%). The rate of extra-intestinal manifestations was 3.6% (95% CI: 2.8-4.5%). Colectomy and colorectal cancer history were found in 14.2% (95% CI: 12.7-15.8%) and 0.5% (95% CI: 0.3-0.9%) of patients, respectively. The most common treatment was 5-ASA (82.9%, 95% CI: 81.2-84.5%), followed by immunomodulator, prednisolone, and anti-TNF (38.4%, 95% CI: 36.3-40.6%). Among immunomodulator users, 90.4% received azathioprine, 4.6% received 6-mercaptopurine, and 5% received methotrexate. Among anti-TNF users, 71.9% received adalimumab (CinnoRA®), and 28.1% received infliximab (Remicade®). Table 2 shows IBD patients’ phenotype, disease course, and outcome.

## Discussion

 In this study, the prevalence of UC was higher than CD, which is in line with the previous report from 2012,^27^ and is similar to the rest of Asia and Western countries.^28^ This study is the first report on the natural history of IBD, including behavior, clinical outcome, and medication usage from Iran as a representative population of the Middle East, which is among the two most populous countries in this region.

 In this study, we observed one peak at 30-40 years, which is similar to previous reports from other countries in Asia.^16^ However, two age peaks have been reported in patients in Western countries (CD: 20 to 30 and 60-70; UC: 30 to 40 and 60 to 70).^15^ This finding could be related to the colorectal cancer screening colonoscopies that are more commonly conducted in Western countries. The lower screening rate in our country may contribute to the underdiagnosis of asymptomatic IBD in older adults.

 In this study, we observed male dominancy in CD patients with a male-to-female ratio of 1.28. Gender distributions differ across geographic regions of the world and by age.^29^ While data from North America,^30-32^ Scandinavia^33^ and Europe^34^ show greater female incidence compared to males, the reverse has been reported from Eastern countries with male to female ratios ranging from 1.5 to 3.3.^28^ These geographical differences raise speculation that there may be genetic and environmental factors playing a role in the pathogenesis that need further investigation.

 In this study, most UC cases had pancolitis followed by left colitis and proctitis. Among Southeast Asian patients, pancolitis has been the most common UC extent (39.5%; range, 28%–56%), followed by left‐sided colitis (37%; range, 22%–58%).^28^ In a meta-analysis, pancolitis was the predominant location of disease in the USA (57.69-60.72%), and proctitis was the least common (8.82 and 8.53%).^35^ Reports from the Middle East show extensive colitis predominance (42.7%–45.5%) in Lebanon and Saudi Arabia.^36,37^ However, in Qatar and the UAE, left-side colitis was the most common UC extent (48%–55%).^6^ Similarly, left-sided colitis was dominant (50%) in a report from Western Hungary^38^ and Brazil.^39^ And, data from Scandinavia show an even distribution of UC extent.^40^ In summary, the extension of UC patients is comparable between Asian countries and the West.

 Regarding disease location in CD patients, in this study, ileocolonic was the most common type (43.7%), followed by ileal and colonic. The Western literature demonstrates relatively equal proportions of CD patients with ileal, colonic, or ileocolonic involvement. In a meta-analysis from the USA, the distribution of CD location was 42% ileocolonic, 28% ileal, and 28% colonic.^35^ A European Collaborative Study Group on Inflammatory Bowel reported 47.4% colonic, 33.9% ileocolonic, and 18.6% ileal.^41^ Data from Scandinavia shows 49% colonic involvement followed by 28% ileal and 23% ileocolonic.^40^ However, ileocolonic involvement is the most common phenotype observed in 50-70% of CD patients in East Asia.^17-20^ In summary, the clinical presentations of CD patients are comparable between Asian countries and the West.

 Among Asian patients, fistulizing behavior ranges 7-18%, and stricturing behavior has been reported between 8-32%.^28^ A meta-analysis calculated 27.7% fistulizing and 16.8% stricturing behavior in the USA,^35^ And data from Scandinavia show 10% fistulizing and 13% stricturing behavior.^40^ In this study, the prevalence of stricturing behavior was only 4.6%, which is lower than other reports, while the prevalence of fistulizing behavior (11%) was similar to Asian countries and lower than the West. In this regard, one contributing factor may be that 3D imaging is not commonly used in Iran, and access to MR enterography, CT enterography, and transrectal EUS is limited. Therefore, underdiagnosis may lead to a lower prevalence of fistulizing and stricturing behavior observed in this study.

 Reported frequencies of extra-intestinal manifestations in IBD range from 6% to 47%, with a frequency of 10-25% in Western countries.^42^ Observed frequency of 5.2% in patients with UC and 3.6% among patients with CD highlights the importance of comprehensive examination of patients and the need to include this topic in the continuing medical education (CME) programs. In addition, this study gathered data from the gastroenterologists, and due to the lack of universal electronic health care records, it could be possible that these patients had records of extra-intestinal manifestation in their charts with other specialties (rheumatologist, dermatologists, ophthalmologists, etc) that were missed.

 Globally, the colectomy rate and colorectal cancer in IBD patients has decreased over the past decade,^43,44^ and the use of biological medications has led to lower need for surgery. Previously, a matched cohort study in America revealed a higher incidence of rectal tumors among UC but not CD patients,^45^ and a large study from England reported the prevalence of colorectal cancer to be 1.3% among patients with IBD.^46^ Our study showed a prevalence of 0.3% for colorectal cancer among patients with UC. It is important to interpret these estimates for surveillance strategies. Of note, higher reported rates in Western countries could also be attributed to better documentation, and future studies should investigate the IBD registry and the cancer registry database together to account for IBD patients who presented with colorectal cancer and underwent colectomy. However, the difference in genetic and environmental factors may also play a role that needs further investigation.

 The usage of anti-TNF and biological agents in this study was lower compared to Western countries, which could be attributed to the limited insurance coverage and lack of access to other types of biologics and small molecules (Infliximab, Adalimumab and Tofacitinib are the only available medications in Iran). Our team, as the focal point of physicians’ and patients’ education on IBD, has been organizing workshops in each province of Iran to increase the knowledge of physicians and patients about the treatment modalities and biologic drugs to start treatment in early stages and prevent morbidity due to cancer, colectomy, stricture or fistula. Adalimumab was used more than infliximab among both UC and CD patients, which could be due to the easier process of commencing (subcutaneous vs. intravenous), considering the more limited access to intravenous injections in rural areas. The high prevalence of 5-ASA usage among CD patients in our cohort is a sign of malpractice, and since CD is a transmural disease and 5-ASA does not have an established role in CD treatments, there is a need for a change of practice. In this regard, there are active plans for incorporating this topic in CMEs. The limited use of methotrexate in this cohort, as well as its roles in decreasing antibodies against biological medications, disease control, and lower cost, highlight the need for the education of physicians to leverage this option in the treatment of patients.

 The referral nature of our centers in this study can lead to selection bias; however, we have included the majority of gastroenterologists in all the provinces of Iran. Moreover, the participation rate was different by provinces in the IRCC. While part of the data extraction was based on clinical records, the research assistant asked retrospective questions from the patient, which might have contributed to recall bias. Moreover, reported medication use in our study was determined by the history of any IBD-related medication intake. Furthermore, this study did not investigate the length and dose of medications.

## Conclusion

 In this national registry-based study, there are significant differences in some clinical phenotypes such as the prevalence of extra-intestinal manifestations and treatment strategies such as biological use in different geographical locations. More inclusive epidemiological studies are needed to characterize patients with IBD from underrepresented populations to reduce the disease burden worldwide.
